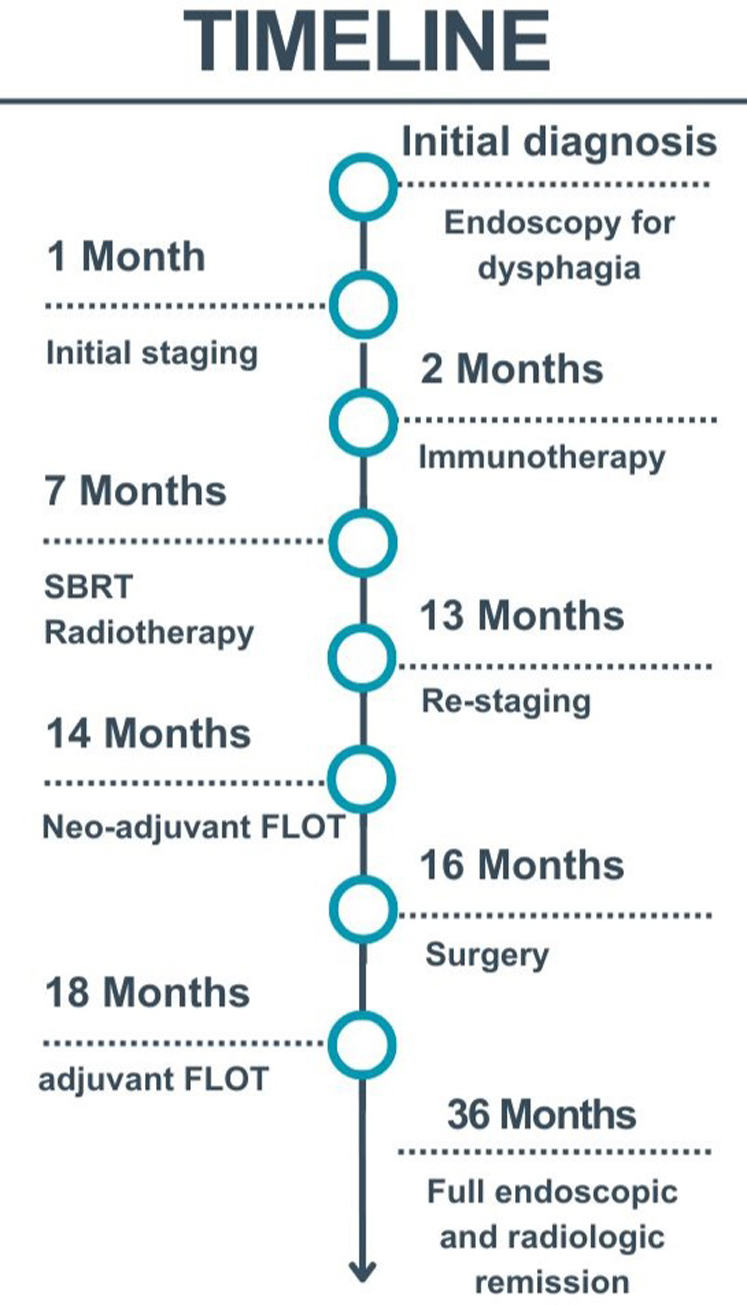# Synchronous Minimally Invasive Resection of Carcinomas of Lung and Esophagus After Downstaging by Palliative Immunotherapy

**DOI:** 10.1016/j.atssr.2025.01.024

**Published:** 2025-02-26

**Authors:** Robbe Van Dyck, Bob Edon, Thierry Wagner, Sven Philippi, Flaviu Crisan, Georges Decker

**Affiliations:** 1 Department of Thoracic Surgery, Hôpitaux Robert Schuman Luxembourg, Luxembourg; 2 Department of Medical Oncology, Hôpitaux Robert Schuman Luxembourg, Luxembourg; 3 Department of Thoracic Oncology, Hôpitaux Robert Schuman Luxembourg, Luxembourg; 4 Centre François Baclesse. National Radiotherapy Center of Luxembourg, Esch-sur-Alzette, Luxembourg; 5 National Center of Pathology, Laboratoire National de Santé, Luxembourg, Dudelange, Luxembourg

## Abstract

We report a case of an elderly patient with synchronous locally advanced bilateral lung and esophageal adenocarcinomas downstaged by atypical multimodality induction therapy. A short course of palliative immunotherapy (stopped for complications after 2 cycles), followed by stereotactic lung irradiation and later neoadjuvant fluorouracil, leucovorin, oxaliplatin, and docetaxel (FLOT) chemotherapy, allowed subsequent synchronous minimally invasive combined esophagectomy and anatomical lung resection with excellent outcome. This report further illustrates the technical feasibility of minimally invasive synchronous anatomical lung and esophagus resection after multimodal therapy, including immunotherapy.

Localized synchronous lung and esophageal carcinomas are challenging, especially in elderly patients. When both cancers are locally advanced, combined surgical treatment is rarely possible because of high surgical risks. Palliative treatment with immunotherapy can offer major local responses and could potentially prolong survival in patients with programmed death ligand 1 (PDL-1) tumor expression. However, the exact potential of immunotherapy for rendering inoperable patients operable is still unknown, and the feasibility of major combined resections in this setting has not been reported.

A 78-year-old man with smoking history presented to the emergency department with recent onset dysphagia. Gastroscopic biopsy confirmed a locally advanced adenocarcinoma of the gastroesophageal junction (GEJ) cT3 cN1 cM0, and staging revealed synchronous bilateral lung adenocarcinomas stage IVa (*TNM Classification of Malignant Tumours*, *8th Edition*), cT2 cN1 cM1a (right lower lobe tumor of 43 mm confirmed by computed tomographic-guided biopsy and left upper lobe tumor of 32 mm) with an additional left lower lobe nodule (7-mm diameter in segment 6) (Figure 1, Supplemental Figure 1).

**Figure 1:**
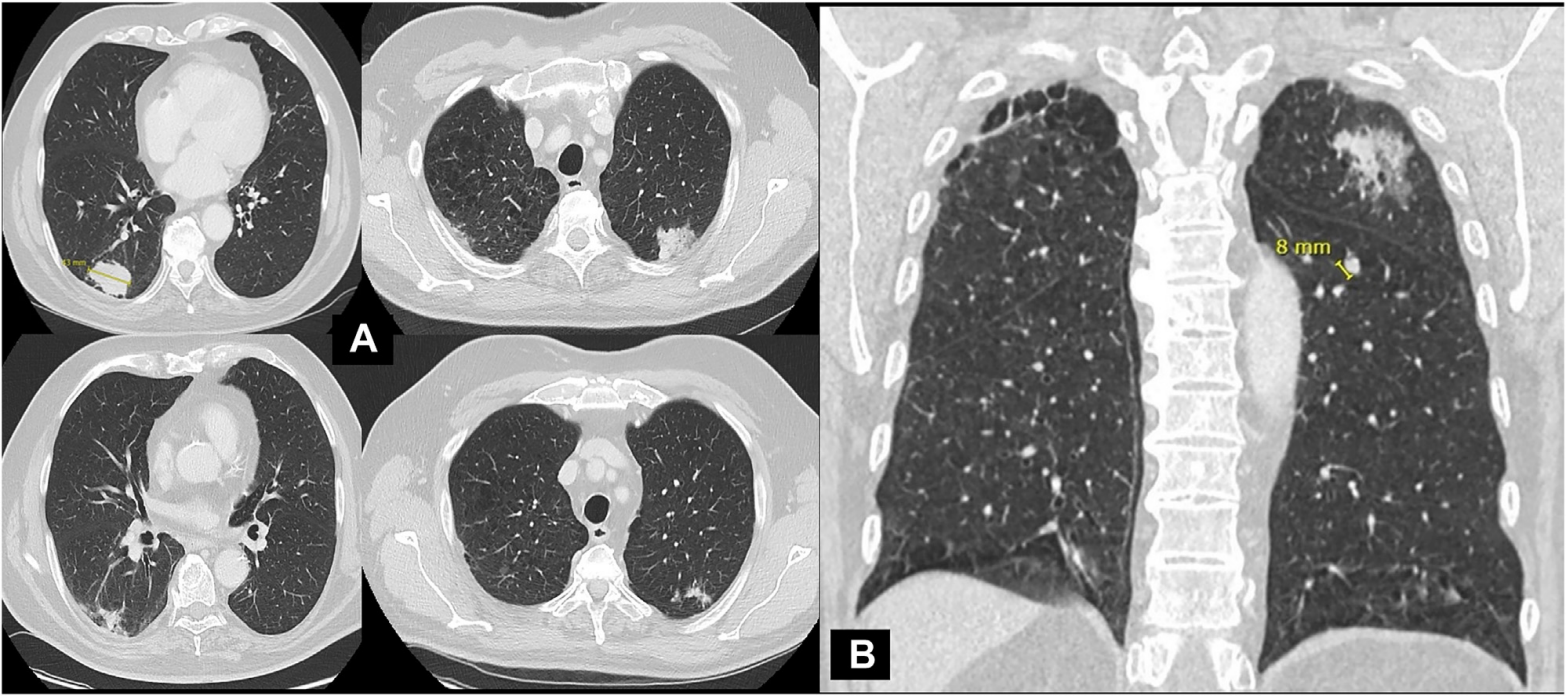
Computed tomography scans show (a) situation before (above) and after (below) 6 weeks of immunotherapy of right lower-lung and left upper-lung tumor with massive response of both tumors after immunotherapy and (b) frontal view of left upper-lung tumor and S6 nodule before immunotherapy.

The patient was considered incurable by thoracic and digestive tumor boards. There was no microsatellite instability in the GEJ tumor and no targetable mutation in the lung adenocarcinoma, but significant PDL-1 expression in both cancers (GEJ: Combined Positive Score, 20; lung: PDL-1 expression, 90%). A palliative immunotherapy was proposed. Pembrolizumab (Keytruda [Merck & Co, Inc], 200 mg, intravenous, 30 minutes, every 3 weeks) was started but poorly tolerated (grade 3 myositis and hypothyroidism) and stopped again after only 6 weeks (2 cycles) (Figure 1). Reassessment showed that by that time the left upper lung tumor had already vanished. Stereotactic body radiotherapy of the right lower-lobe tumor (5×12 Gy) was administered, resulting in local tumor control.

Gastroscopic reevaluation showed an unchanged Siewert II tumor with endoscopic ultrasound uT3 N1 aspect (Figure 2c). Lung tumors on both sides remained regressed on computed tomographic scan and positron emission tomography. The initial maximum standard uptake value of 10.0 of the right lower-lobe tumor decreased to 3.7 at restaging. The GEJ tumor remained negative on positron emission tomography imaging (Figure 2). The left lower-lobe nodule was unchanged (7 mm) and thus presumed to be benign. The result of mediastinal nodal staging by endobronchial ultrasound was negative.

**Figure 2:**
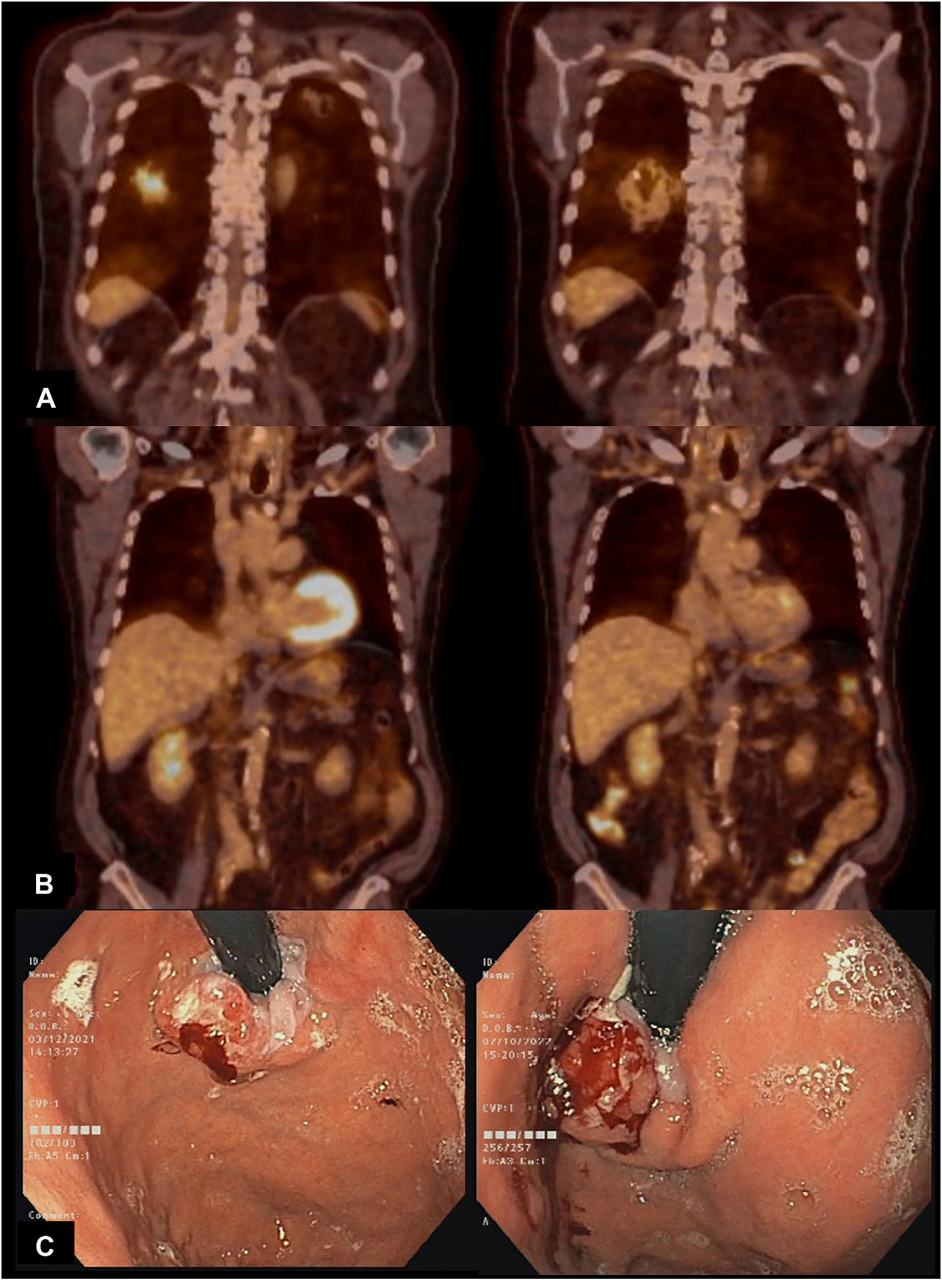
Initial staging and restaging 13 months after initial diagnosis: (a) right lower-lobe tumor before immunotherapy (left) and after immunotherapy and stereotactic body radiotherapy (right). (b) Positron emission tomography scan of gastroesophageal junction tumor shows no significant hypermetabolism before (left) or after induction therapy (right). (c) Gastroscopy at the initial diagnosis (left) and 13 months later before neoadjuvant fluorouracil, leucovorin, oxaliplatin, and docetaxel (FLOT) chemotherapy (right).

In this situation, 14 months after the initial diagnosis, 4 cycles of neoadjuvant fluorouracil, leucovorin, oxaliplatin, and docetaxel (FLOT) chemotherapy were administered every 2 weeks at 50% dose reduction: fluorouracil, 2600 mg/m^2^; leucovorin, 200 mg/m^2^; oxaliplatin, 85 mg/m^2^; and docetaxel, 50 mg/m^2^. The patient remained fit (Eastern Cooperative Oncology Group score 0, American Society of Anesthesiologists Physical Status Classification score II, and body mass index of 28 kg/m^2^) and underwent combined single-stage surgery.

By a fully minimally invasive approach in 1 session, first a video-assisted thoracoscopic surgery wedge resection of the left segment 6 nodule confirmed a benign hamartoma. Then a laparoscopic and right thoracoscopic Ivor Lewis minimally invasive esophagectomy (MIE) with right lower lobectomy and 2-field lymph node dissection was done (Figure 3). Total operative duration was 640 minutes (right video-assisted thoracoscopic surgery, 324 minutes) and total blood loss was 250 mL. The patient was extubated on-table.

**Figure 3:**
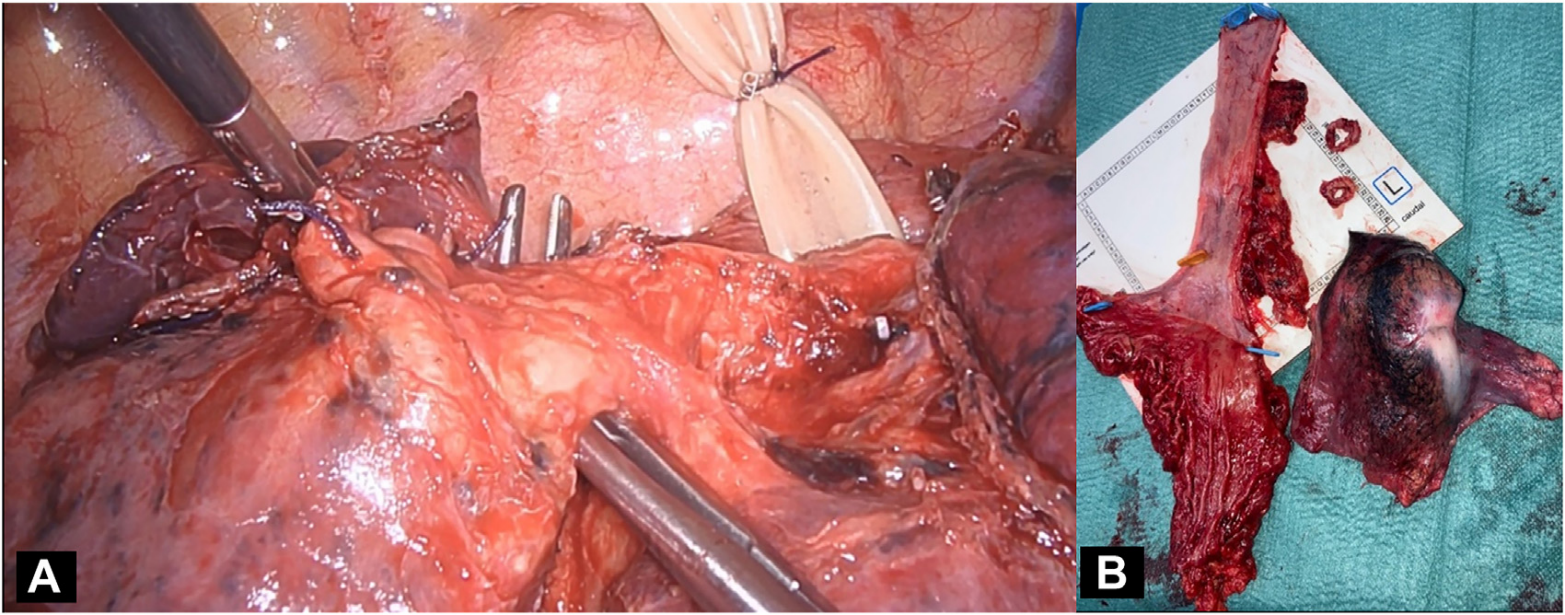
(a) Intraoperative view during simultaneous video-assisted thoracoscopic right lower lobectomy (clamp around right lower-lobe bronchus) and mobilized esophagus (inside penrose). (b) Specimens of Ivor Lewis minimally invasive esophagectomy and right lower lobe.

Owing to transient left vocal cord paresis, oral renutrition was delayed until day 8. No other complications occurred (Clavien-Dindo score 1), and the patient was discharged home at day 19. He recovered to complete 4 cycles of adjuvant FLOT (dosage reduced to 50%, every 2 weeks), starting 4 weeks postoperatively.

Final pathology showed residual distal third adenocarcinoma (1.5×1.2 cm entirely located above Z-line) with major response (modified Ryan score): ypT2 N0 M0, R0, G3, LV1, PN0, 0 of 41 lymph nodes and subcarinal signs of nodal treatment response. The right lower-lobe mass of 60×55 mm showed several 1- to 2-mm foci of residual viable adenocarcinoma (ypTis N0 M0, R0).

The patient fully recovered with follow-up showing no recurrence 20 months after surgery, respectively 36 months from the initial diagnosis. A timeline of this case, including diagnosis and treatment steps, is provided in Supplemental Figure 2.

## Comment

Our case confirms that immunotherapy can dramatically alter the evolution of synchronous locally advanced lung and esophageal adenocarcinomas. The beneficial downstaging allowed this patient to undergo curative surgery even though immunotherapy had to be limited to 2 injections because of toxicity.

Reports of combined esophageal and anatomical lung cancer resections are sparse, and this combination may increase morbidity mainly by increased esophageal anastomotic fistulas.^1^ This might even more be a risk in elderly patients where esophagectomies alone already carry increased operative risks.^2^ Preoperative immunotherapy is known to increase the technical difficulties of subsequent anatomic lung resections.^3^ In our case, stereotactic body radiotherapy further added to the complexity of combined Ivor Lewis MIE with lower lobectomy and adequate lymphadenectomies.

Only 2 case series combining these 2 major operations have been reported to date. Several major oncologic centers in North America collected 18 patients over a 22-year period.^4^ In China, Liu and colleagues^1^ combined data from several “ultra-high volume” centers. However, none of the patients in either series underwent minimally invasive surgery. Very few patients had received any induction therapy, with none of them having had preoperative immunotherapy. Before our report, only 1 case report has described a minimally invasive approach for combined esophageal and lung resection but without any induction therapy or follow-up.^5^ We believe that besides the role of atypical systemic induction, the surgical outcome of our case was largely aided by the minimally invasive nature of the surgery.

In conclusion, even abbreviated immunotherapy can occasionally downstage lung adenocarcinomas sufficiently to pass from a palliative to a potentially curative setting. Synchronous MIE esophagectomy combined with major lung resections can be feasible after multimodality therapies, including immunotherapy.

## Figures and Tables

**Supplementary Figure 1:**
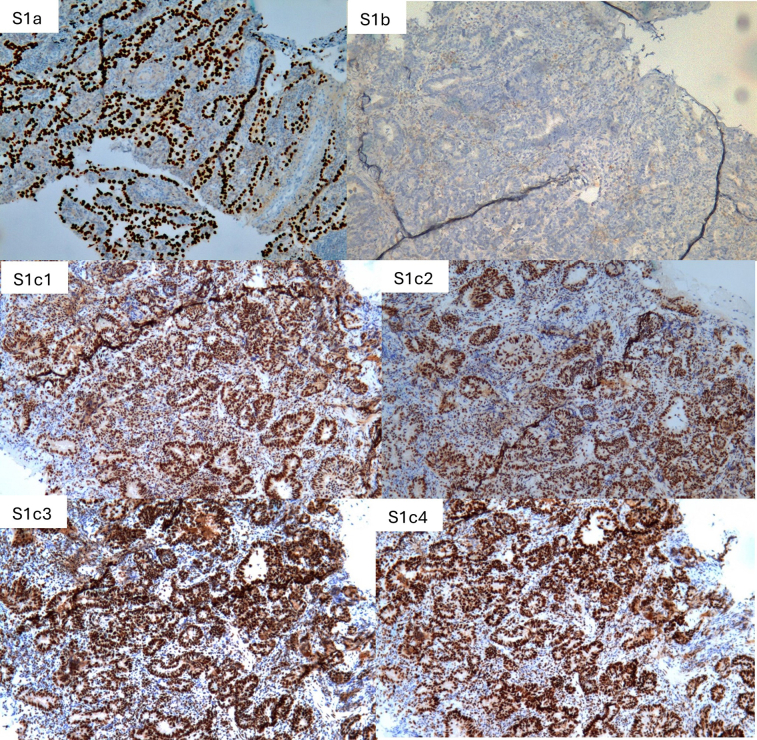


**Supplementary Figure 2:**