# Supplementary material for: Synchronous Minimally Invasive Resection of Carcinomas of Lung and Esophagus After Downstaging by Palliative Immunotherapy
Source: Ann Thorac Surg Short Rep. 2025 Feb 26;3(3):749–53. doi: 10.1016/j.atssr.2025.01.024 (PMC12559253; doi:10.1016/j.atssr.2025.01.024)
Supplement: Supplementary Material [file mmc1.docx]

**SUPPLEMENTARY MATERIAL**

Supplementary Fig. S1: Pathological slides of lung biopsies and esophageal cancer biopsies showing a primary adenocarcinoma of the right lower lobe (TTF-1 positive) as well as a primary adenocarcinoma of the gastroesophageal junction. (S1a) TTF-1 positive lung adenocarcinoma. (S1b) HER2 negative esophageal adenocarcinoma. (S1c) Immunohistochemistry stains performed on esophageal adenocarcinoma tissue suggesting absence of microsatellite stability of the tumor. (S1c1) stain positive for PMS2. (S1c2) stain positive for MLH1. (S1c3) stain positive for MLH2 (S1c4) stain positive for MSH6.

Supplementary Fig. S2: Timeline of patient course and treatment.
